# Mother’s fibula in son’s forearm: use of maternal bone grafting for aneurysmal bone cyst not amenable to curettage – a case report with review of literature

**DOI:** 10.1051/sicotj/2015043

**Published:** 2016-04-20

**Authors:** Mohammed Tahir Ansari, Deepak Gautam, Prakash P. Kotwal

**Affiliations:** 1 Department of Orthopedics, All India Institute of Medical Sciences (AIIMS) 110029 New Delhi India

**Keywords:** Aneurysmal bone cyst, Maternal bone grafting

## Abstract

It has always been a challenge to reconstruct large bone gaps. The aim of this case report is to highlight the success of homologous maternal bone grafting in a large cystic lesion. A six and half years old boy presented to us with an aneurysmal bone cyst (ABC) of the right radius, not amenable to curettage. We excised the lesion in toto, which created an 11 cm bone loss. Considering the age of the patient, we reconstructed the bone gap with maternal fibular graft. Accordingly, 12 cm of fibular graft was harvested and fashioned to fit into the bone gap. It was fixed with an intramedullary K-wire. No cancellous graft was used in the procedure. The limb was kept in the above elbow cast till incorporation of the fibula was noted on the radiographs. Six months following surgery the skiagram showed that the fibula was incorporated. Mobilization of the elbow and wrist was started along with strengthening of the forearm muscles. K-wire was removed at nine months. At the latest follow up of 24 months, the fibula is fully incorporated, the child regained full range of motion and strength of elbow. We discuss the techniques adopted in this particular case along with the review of literature.

## Introduction

Aneurysmal bone cyst (ABC) is a rare non-neoplastic expansile osteolytic bone lesion of unknown etiology. Aneurysmal and solitary bone cysts develop most commonly during skeletal growth. The annual incidence of primary aneurysmal bone cyst is 4 per 100 individuals. The male to female ratio is 1:1.04, and the median age is 13 years (range, 1–59 years) [1]. In a separate study by Zehetgruber et al., the annual prevalence was 0.32 per 100,000 individuals (range, 0–1.238) for aneurysmal cysts, with a 1.8:1 male to female ratio and a median age of the patients of 11.1 years (range, 1–19.7 years) [2]. Although benign, the ABC can be locally aggressive and can cause extensive weakening of the bony structure and also impinge on the surrounding tissues. Curettage with or without bone grafting has remained as the mainstay of treatment [3]. Recurrences are not uncommon. The rate of local recurrence after this procedure has been reported to range from 10% to 59% and even up to 70% in children below 10 years of age [4–6].

We report a case of homologous maternal bone grafting after excision of a large benign cystic lesion involving the right radius bone in a boy of six and half years which was not amenable to curettage. We suggest that maternal fibular allograft is a possible option for treating a large gap after resection of a bone cyst in a pediatric age group.

## Case report

**Figure 1.**
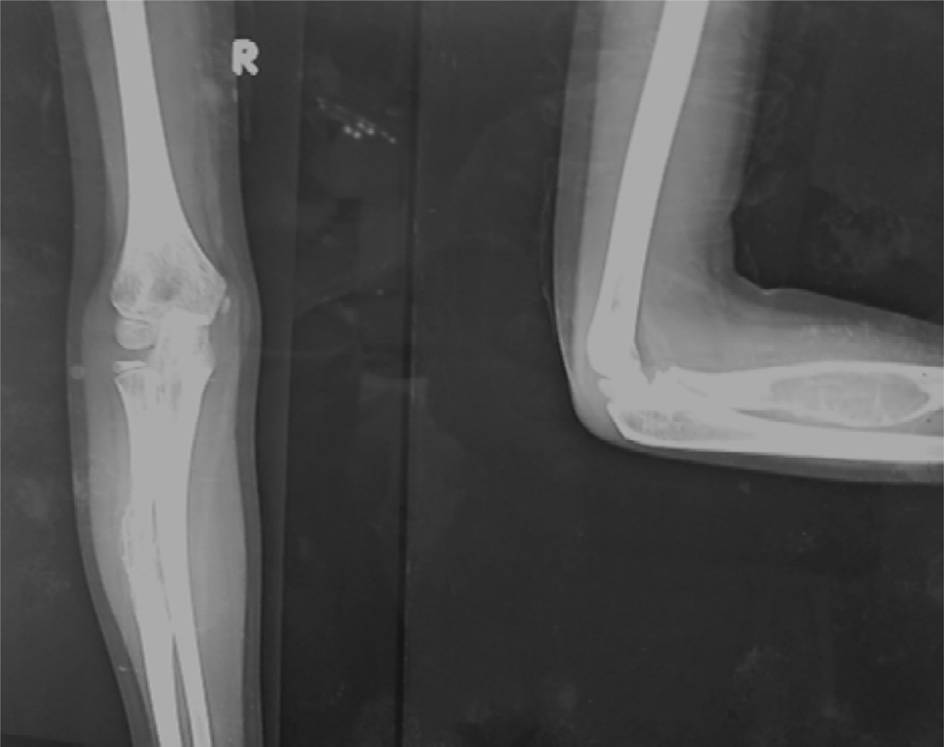
Initial anteroposterior and lateral views of roentgenograph showing an expansile lytic lesion in proximal half of the radius.

**Figure 2.**
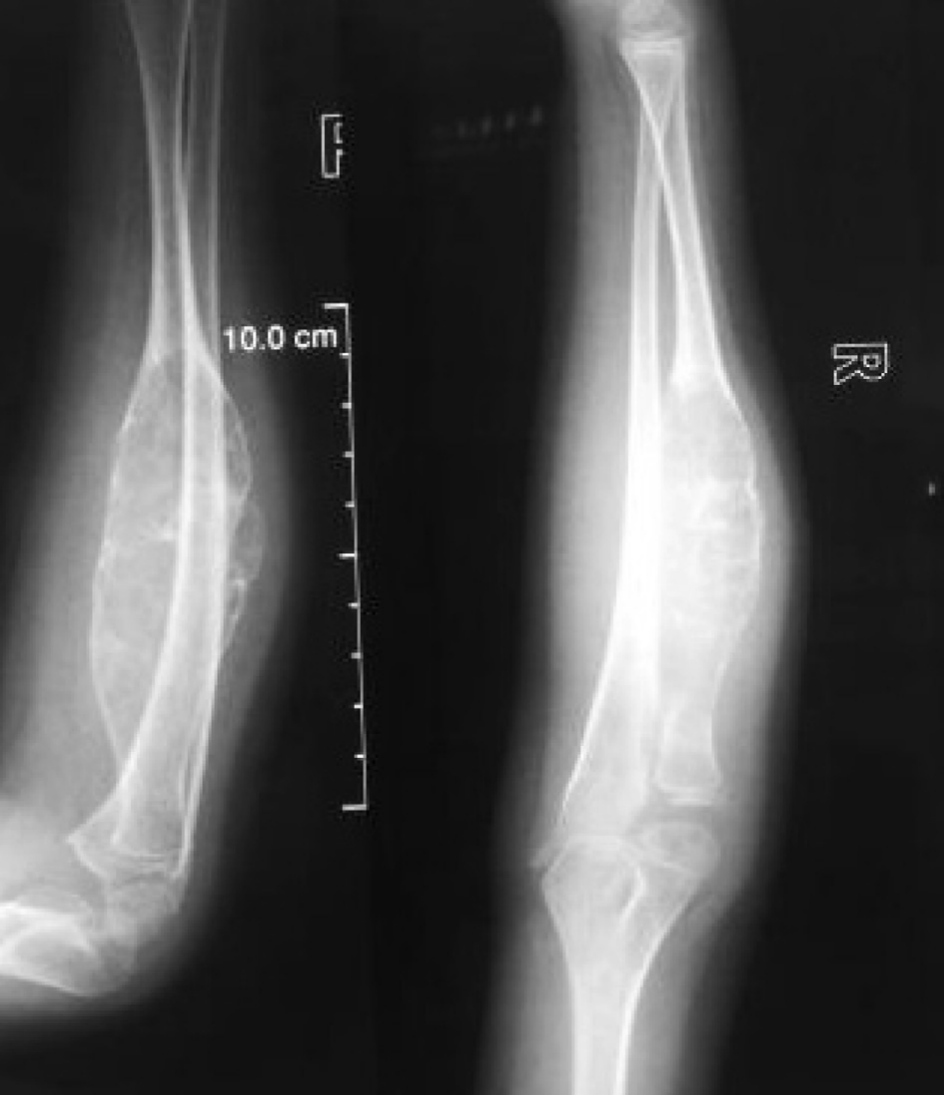
Anteroposterior and lateral views suggesting that the lesion had increased in its size with thinning out of cortex.

**Figure 3.**
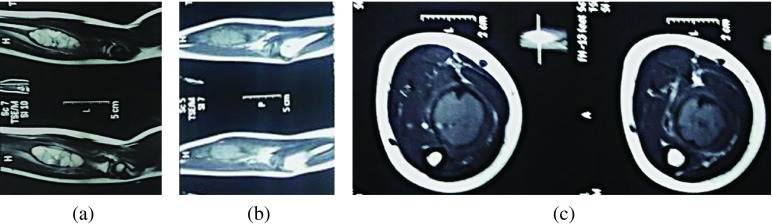
(a) T2 weighted sagittal MR images showing meta-diaphyseal expansile lesion with multiple hyperintense cysts with fluid-fluid levels and surrounding complete hypointense rim, (b) T1 weighted sagittal MR images showing iso to hypointense lesion, (c) T1 weighted sagittal MR images showing well marginated expansile iso to hypointense lesion.

A six and half year old boy referred to us from another center had complaints of an insidious onset of pain in his right forearm, which initially was less intense. The parents ignored it initially thinking it might be due to his excessive playful nature and cycling. They had a delay of three and half months to consult a doctor in their town when the child complained of an increasing pain. In the meantime, there appeared some swelling in his forearm, which was gradually progressing. A X-ray was done (Figure 1) which revealed an expansile lytic lesion in the proximal half of the radius. He was initially kept under observation and was then referred to a higher center. A repeat X-ray was done, which revealed an expansile lytic lesion involving the proximal half of the radius. On comparing the previous X-rays (Figure 2), it was seen that the lesion had significantly increased in size with thinning of the cortex rendering it difficult to decide the treatment options. As per the protocol, an MRI was done (Figures 3a–3c) which revealed low to intermediate signal intensity with a rim of low signal intensity with internal septa. A bone biopsy was done which after radiological correlation was confirmed to be a case of aneurysmal bone cyst.

### Operative procedure

The tumor was expansile with papery thin cortices and hence was not amenable to curettage. It was decided to do wide resection of the tumor. Reconstruction of the radius following tumor resection can be done with different techniques. First to use child’s own fibula may be considered as vascularized or nonvascularized pattern. Second, the cadaveric allograft. Third, an allogenic allograft from the mother. The parents were informed regarding all the options for reconstructing the radius following excision of the tumor. We were a bit concerned with regard to the diameter of child’s own fibula to be used with the radius because of the possible size mismatch as well as the surgical trauma at two sites. The parents were reluctant to use the allograft stored in the bone bank and hence opted for the mother’s fibula. A written consent was taken from both the parents. Two operating tables were used with two surgical teams. The mother’s surgery was commenced after exposing the tumor in the child and establishing length. Both the surgeries were conducted under tourniquet to reduce blood loss.

#### Surgery for the child

**Figure 4.**
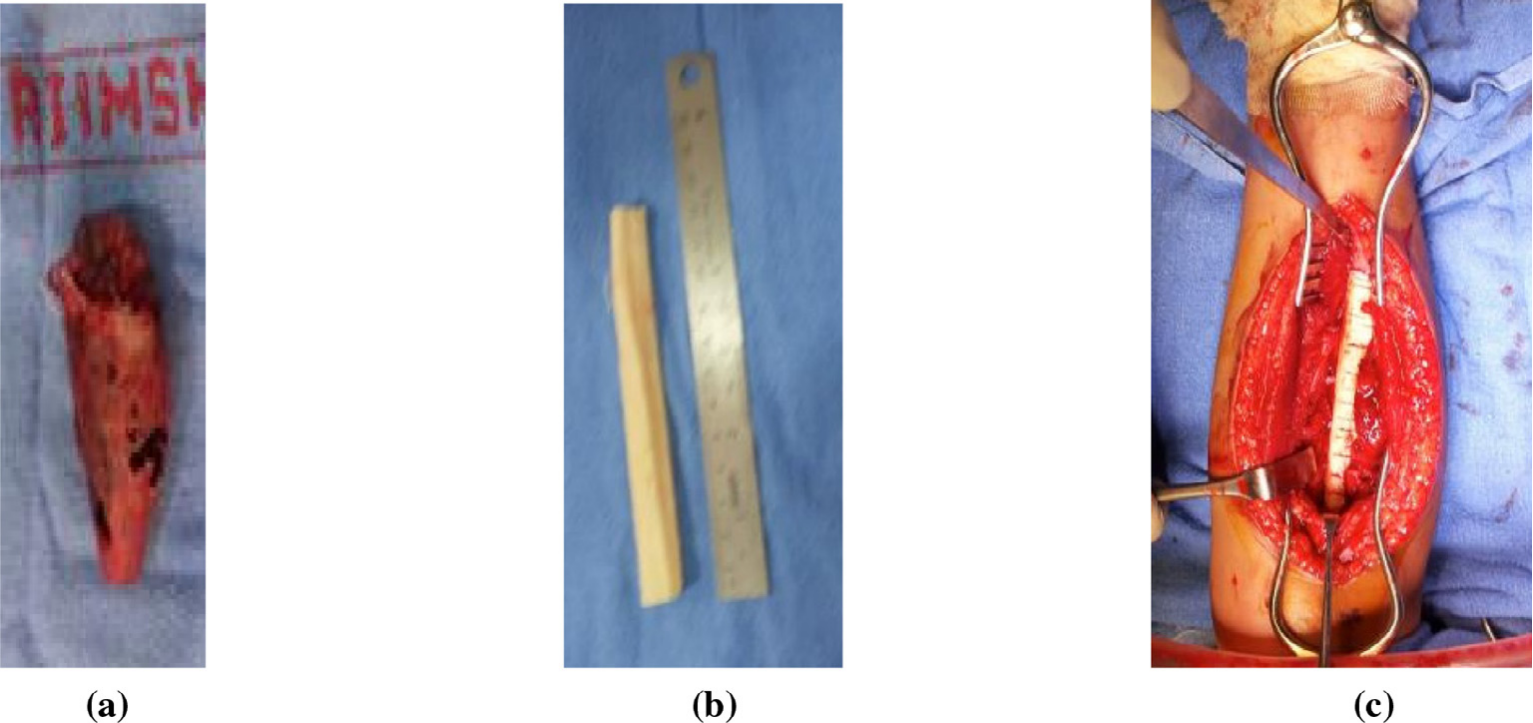
Intraoperative picture showing the (a) resected tumor, (b) harvested maternal fibula, and (c) maternal fibula used for reconstructing the radius.

The radius was exposed using the Henry’s approach with around 15 cm long longitudinal incision along the line joining the lateral side of biceps tendon to the radial styloid process. After incising the deep fascia, a plan was developed between the brachioradialis and flexor carpi radialis. Proximally a plane was developed between the pronator teres and brachioradialis. The superficial radial nerve, radial artery, and poster interosseous nerve all were secured. Periosteum was elevated and was tagged. The exposed tumor length was found to be 11 cm long. The tumor was completely excised (Figure 4a).

#### Surgery for the mother

**Figure 5.**
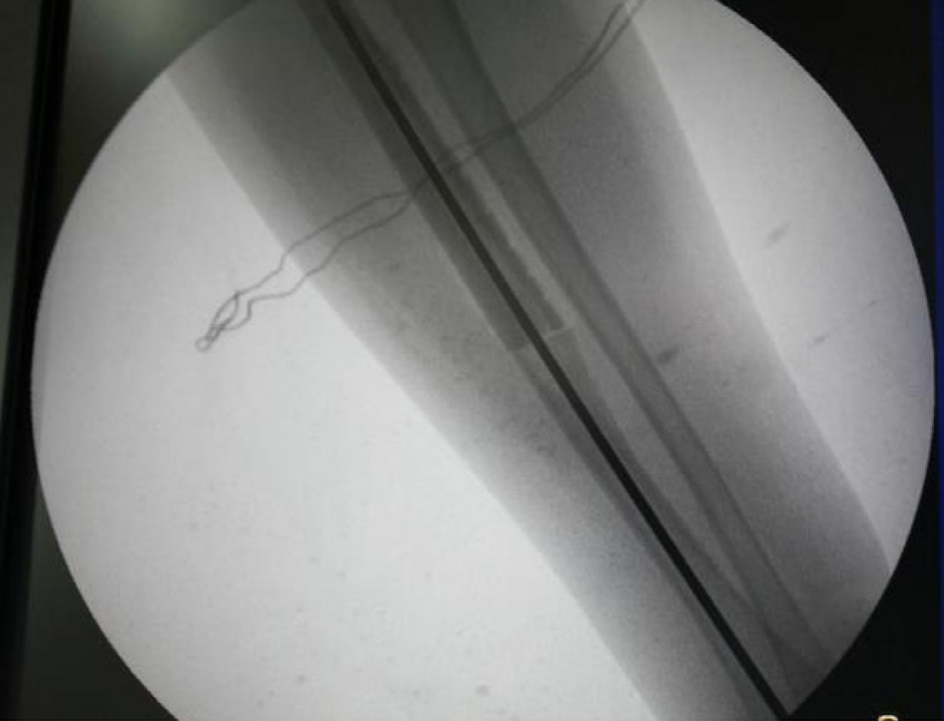
Intraoperative roentgenograph showing reconstruction of the radius using maternal fibular graft fixed with an intramedullary K-wire.

**Figure 6.**
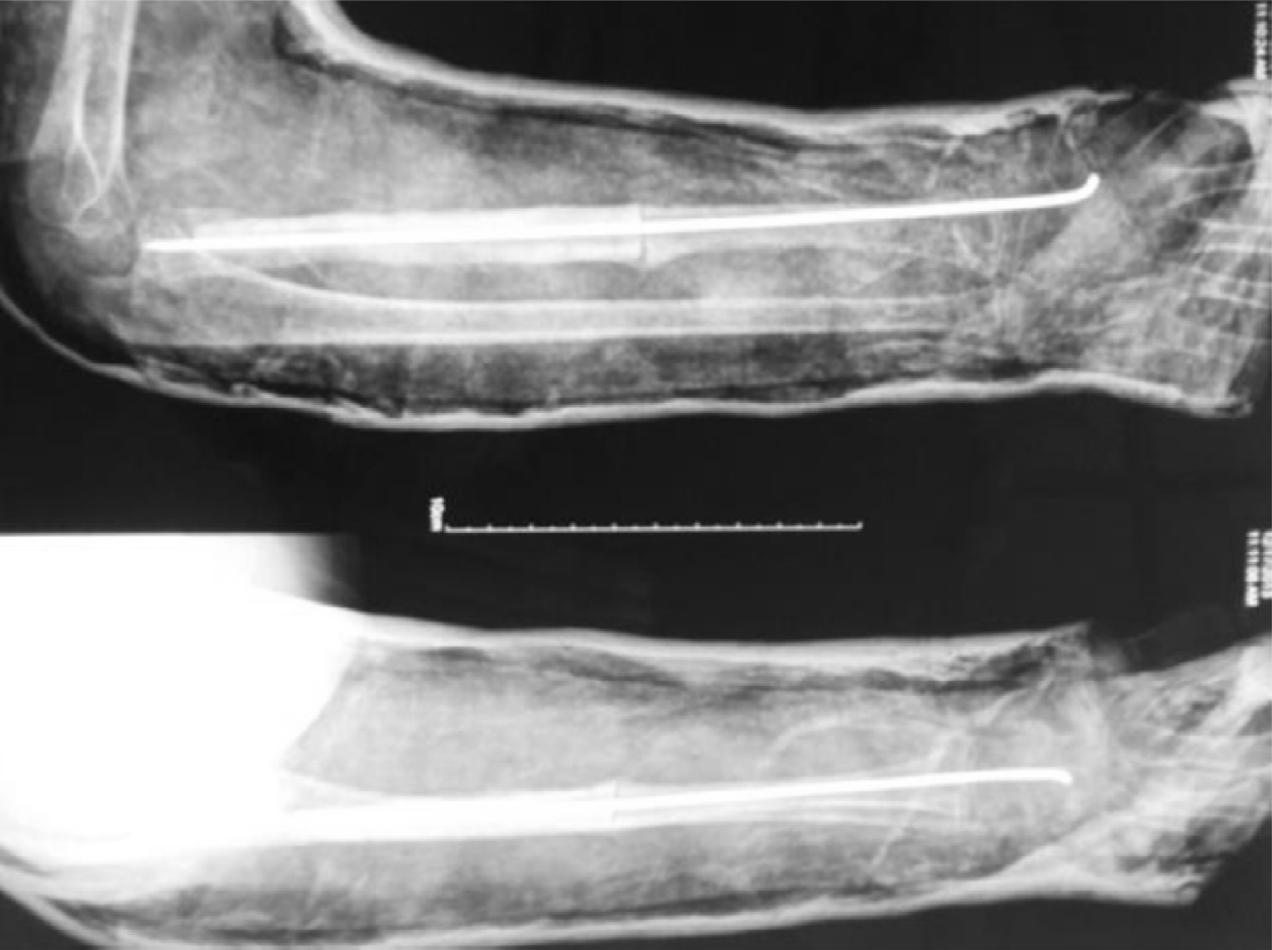
Postoperative X-ray showing reconstruction of the radius using maternal fibular graft fixed with an intramedullary K-wire.

**Figure 7.**
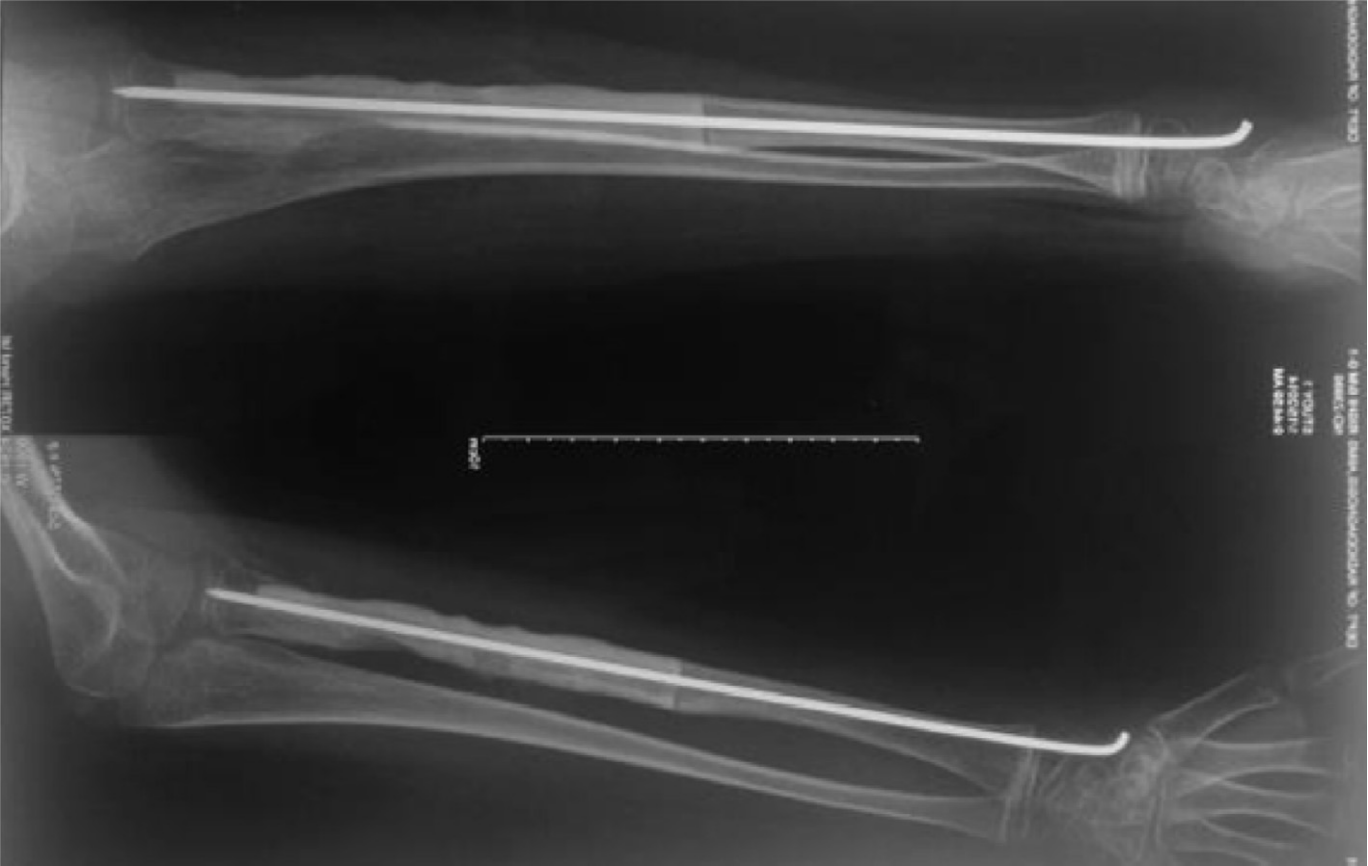
Twelve weeks follow-up X-ray showing incorporation of the graft at the parent-allograft bone interface; resorption of cortical margins is visible. 35 mg of alendronate was started at this time.

**Figure 8.**
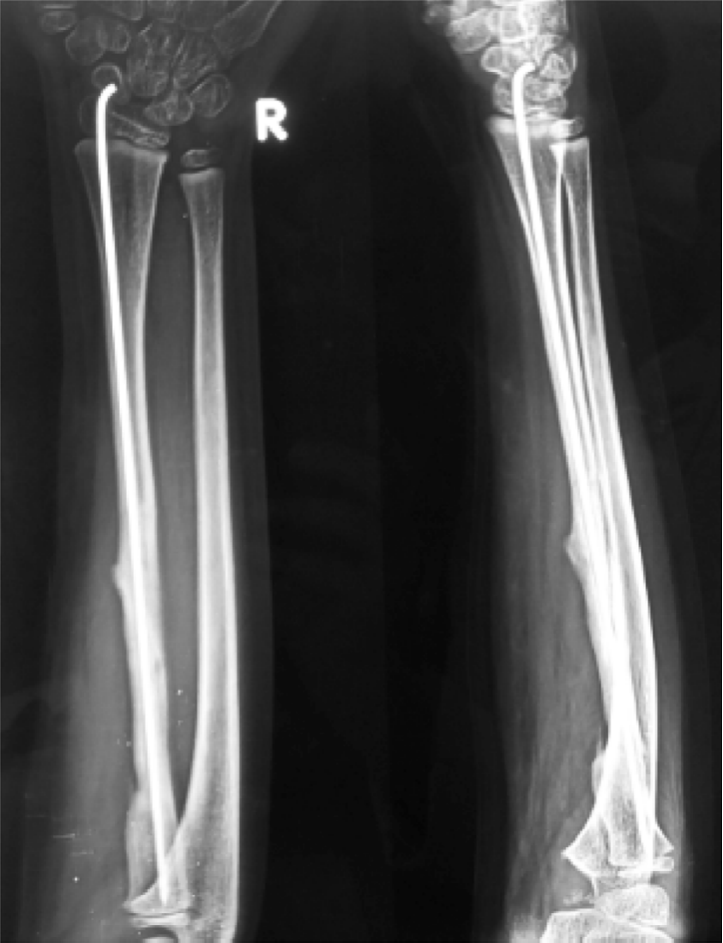
Six months follow-up X-ray showing incorporated graft with K-wire in situ after which the K-wire was removed.

**Figure 9.**
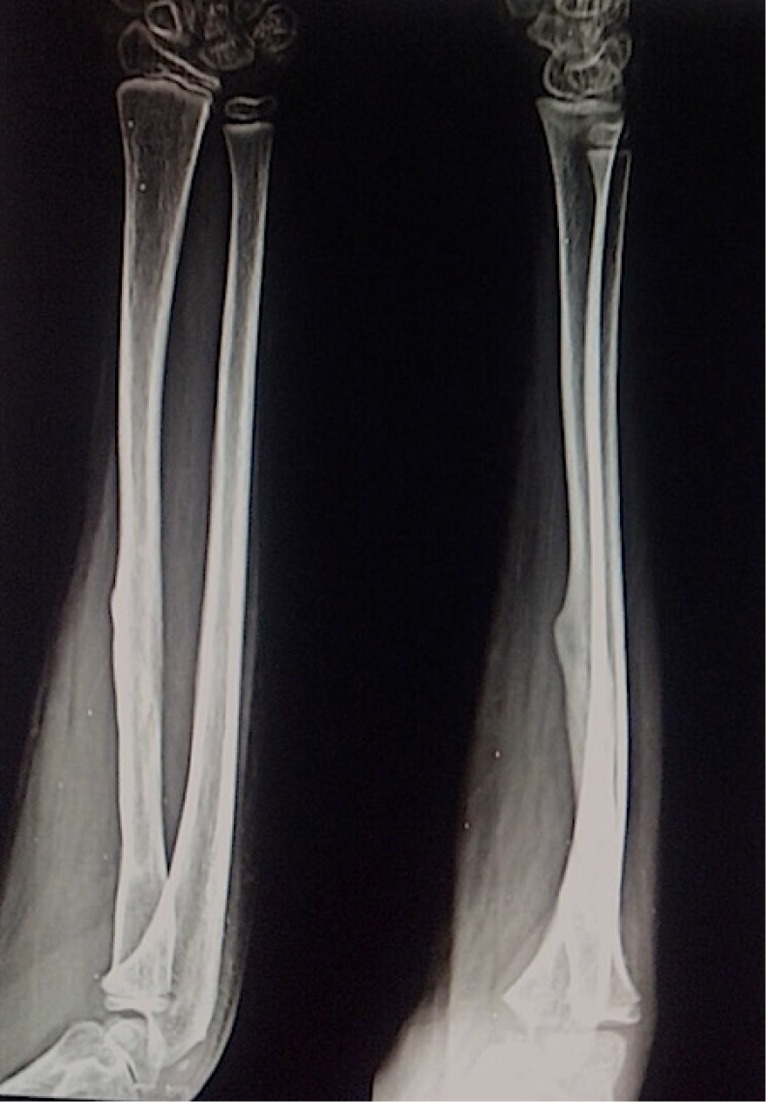
Two years follow-up roentgenograph showing fully incorporated graft into the host bone.

The mother was screened before the surgery and was found to be free of any diseases. It was decided to harvest 12 cm of fibula. A linear incision was made just posterior to the fibula. A plane was developed between the peroneal and the soleus muscles. The muscles as well as the interosseous membrane attached to the fibula were stripped off. The required length of the fibula was taken out (Figure 4b). The harvested fibula was cleared of the medullary contents. The graft was fashioned to fit into the bone gap and small cuts were made over the fibular cortex to facilitate the ingrowth (Figure 4c). It was fixed with an intramedullary K-wire (Figure 5). The periosteal covering was sutured back over the fibular graft. No cancellous graft was used in the procedure. The limb was kept in the above elbow Plaster of Paris (POP) slab in immediate postoperative period (Figure 6). The patient was followed up first at 2 weeks for suture removal. Twelve weeks later following the surgery, there were signs of incorporation of the graft (Figure 7) but some resorption of bone was noticed along the shaft of graft. The patient was started on weekly dose of bisphosphonate therapy (35 mg of alendronate). The cast was removed and a forearm brace was applied. Mobilization of the elbow and wrist was started along with strengthening of the forearm muscles. The roentgenograph at six months (Figure 8) showed significant decrease in bone resorption. The K-wire was removed and the brace discontinued. The bisphosphonate was continued for further three months and stopped. At the latest follow-up of two years (Figure 9), the graft was found to be fully incorporated and the child regained full range of motion and strength of elbow.

## Discussion

Aneurysmal and solitary bone cysts develop most commonly during skeletal growth. The stage 1 ABC can be treated with intralesional curettage [7], the common stage 2 ABC is treated by intralesional excision which involves wide unroofing of the lesion through a cortical window by careful abrasion of all the surfaces with a high-speed burr and, possibly, local adjuvants such as phenol, methyl methacrylate (MMA), or liquid nitrogen [8]. These adjuvants are controversial because firm evidence about their effectiveness is lacking. Moreover, their use entails considerable risk. En bloc or wide excision is typically reserved for stage 3 ABCs that are not amenable to intralesional excision (e.g. extensive bony destruction); the recurrence rate after en bloc excision is only about 7%. Reconstructive options after wide excision include structural allograft and reconstruction with either endoprostheses or allograft-prosthetic composites [9]. Vascularized bone transfer is another option, which requires microvascular surgical expertise. The treatment of a large segmental defect (>6 cm) is challenging and the conventional bone grafting techniques are less reliable for such large defects. The fibula has been widely used to reconstruct long bone defects because of its structural characteristics and low donor site morbidity. Although, the free or vascularized fibular graft is a well-established alternative, with reported good results in forearm reconstruction, its application for such large defects is more complex [10–12]. The prolonged immobilization, difficulty in achieving the stable fixation, and long wait for the hypertrophy of the vascularized fibula are limiting points of its use. In this case, it was decided to use mother’s fibula, as the diameter of the bone would correspond to the resected margin of the child’s radius. In addition; this patient was only six and half years old hence less suitable for contemplating autografts (fibula) for such large defects. Cancellous autograft was not used as the periosteal sleeve was preserved. Mother’s fibula was taken as it has been said that maternally donated recipients have got superior survival. Although the best survival of any type of allograft is said to be from a twin, it was not possible in this case. Tamaki et al. retrospectively compared the outcomes of blood and marrow stem cell transplantation from maternal donors (*n* = 46) to those from paternal donors (*n* = 50). At 5 years, recipients of maternal hematopoietic cells had a significantly higher overall survival than patients receiving paternal grafts (60% vs. 32%, *P* = 0.006) [13]. We could find very little literatures regarding the use of maternal bone graft following tumor resection. Devalia et al. used maternal fibula for the reconstruction of a long-standing segmental non-union of both humeri in a child of three and half years age with osteogenesis imperfecta [14]. At 12 weeks, the humerus was united clinically with radiographs showing callus formation and the fibular allograft was incorporated both proximally and distally completely bridging the defect at final follow-up. Salunkhe and Raiturker have just mentioned the use of maternal fibula as a bone graft in one of his 30 cases in the management of benign lytic lesions of the bone following intralesional excision and marginal resection [15]. It has been mentioned in the literature that the fresh allogeneic bone elicits both acellular and humoral immune response. It helps in the development of enhancing factors that block the detectable immunity and probably protect the graft from rejection. The importance of these observations lies in the potential technique of employing fresh viable allografts; prior freezing and tissue matching for HL-A transplantation antigens are not necessary [16]. In our case, the fibular allograft was cleared of all the bone marrow before it was transplanted into the recipient (son). So, there was no need of antigenicity testing before the procedure. We used bisphosphonate in the middle of follow-up as a precautionary measure so as to prevent the graft from resorption before full incorporation. We could get an excellent outcome at the latest follow-up with our procedure. Unlike autografts, the allografts are not free of the risk of disease transmission. However, meticulous attention was given to screen the mother prior to surgery. She was tested for the presence of viral diseases and viral markers for hepatitis B, hepatitis C, and HIV were done. She was found to be negative for the presence of any disease. The surgery was conducted simultaneously in two tables, so that there was a minimum possible time gap between the graft harvestation and transplantation. All the precautions needed for intraoperative sterility were maintained during the surgery.

## Conflict of interest

The authors declare no conflict of interest in regard with this paper.
